# Graft preservation with multi-stage surgical repair of an aortoesophageal fistula after thoracic endovascular aortic repair – A case report^☆^

**DOI:** 10.1016/j.ijscr.2020.05.073

**Published:** 2020-06-06

**Authors:** Matthias Buerger, Jan Paul Frese, Sebastian Kapahnke, Andreas Greiner

**Affiliations:** Charité – Universitätsmedizin Berlin, Corporate Member of Freie Universität Berlin, Humboldt-Universität zu Berlin, Berlin Institute of Health, Department of Vascular and Endovascular Surgery, Germany

**Keywords:** Aortoesophageal fistula, Thoracoabdominal aorta, TEVAR, Graft infection

## Abstract

**Introduction:**

Aortoesophageal fistula (AEF) after thoracic endovascular aortic repair (TEVAR) is a rare complication associated with high mortality. Most well established treatment is multi-staged surgery, including removal of infected stent graft, esophageal resection and aortic reconstruction.

**Presentation of case:**

We report on a case of a 67-year-old patient with AEF and stent graft infection. Stent removal was infeasible due to the critical condition of the patient and history of multiple vascular procedures of the thoracoabdominal aorta. Surgical management included staged right and left thoracotomy, esophagectomy, vacuum therapy (VAC) on stent prosthesis and subsequent graft coverage with omental and pleural flaps, followed by esophageal reconstruction.

**Discussion:**

An established and generally accepted treatment approach for graft infections does not exist. Graft explantation and radical surgical debridement is the therapy of choice for prosthetic infections. In comparison to previous literature, our case represents the complexity of the treatment of AEF and its enormous demands on the interdisciplinary medical team.

**Conclusion:**

Our report shows that in an emergency situation without other surgical options as in our case, it was possible to stabilize the patient through application of vacuum therapy in the infected area, with simultaneous esophagectomy, followed by secondary staged reconstruction with omentoplasty and pleura parietalis flap remaining the graft in situ.

## Highlights

• Aortoesophageal fistula (AEF) after TEVAR is a rare but challenging complication associated with high mortality rates.
• AEF after TEVAR has to be treated in an interdisciplinary concept.
• If stent removal is not feasible, a multi-stage surgical procedure with leaving the thoracic stent graft in situ can lead to infection remediation.

## 1 Introduction

A standardized therapy regimen for AEF after TEVAR does not currently exist. Possible therapy alternatives include the implantation of esophageal stents, esophagectomy only or in combination with immediate or secondary aortic reconstruction. Standard of care for the reconstruction of the thoracic aorta in case of graft infections is explantation and reconstruction with polyester grafts or replacement by homo- or xenograft. If aortic reconstruction is not possible in an emergency situation, an individual concept must be found. Infection control is the primary therapeutic goal to ensure patient survival. We report a successful outcome of a multimodal staged therapy concept of graft preservation with resection of the esophagus and vacuum therapy as the last resort in a patient with aortic graft infection and AEF after multiple open and endovascular procedures for thoracoabdominal aneurysm (TAA).

This work is reported in line with the SCARE criteria for case report publication [1].

## 2 Case report

**Fig. 1:**
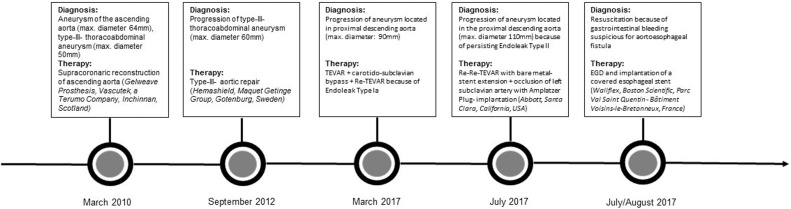
Past medical patient history.

A 67-year-old male was admitted to our emergency department in septic shock after recurrent hematemesis in November 2017. The complex surgical history is shown in Fig. 1. Remarkable is an episode of upper gastrointestinal bleeding, suspected AEF and consecutive esophageal stent implantation in a different institution.

**Fig. 2:**
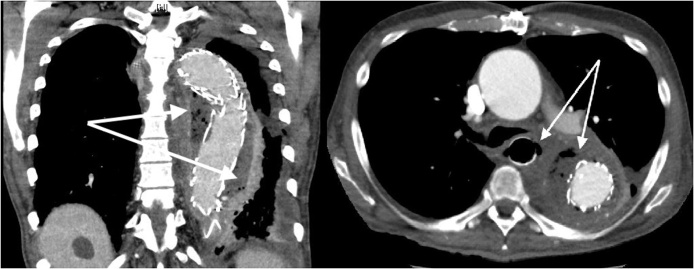
Infection of the former thoracoabdominal aneurysm with paraesophageal air inclusions.

CT scan revealed mediastinitis and aortic graft infection with AEF (Fig. 2).

**Fig. 3:**
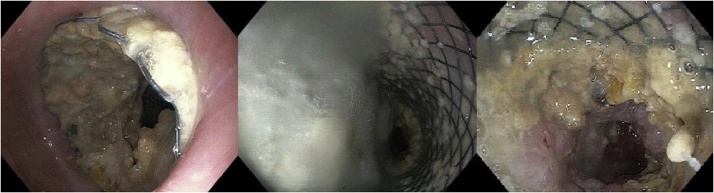
Preoperative esophagogastroduodenoscopy.

Esophagogastroduodenoscopy (EGD) showed a massive accumulation of pus around the esophageal stent (Fig. 3).

As a damage control procedure, right posterolateral thoracotomy, esophageal blind resection and external drainage were performed. Sac of the TAA was opened dorsolaterally from the right. Due to missing signs of leakage the stent graft was left in place. An endoluminal vacuum therapy system (VAC, Eso-Sponge, B. Braun, Melsungen, Germany) was placed in the open sac.

Postoperatively, the patient quickly stabilized under antibiotic therapy. Microbiology showed Candida and Lactobacillus.

Subsequently, a suction-flush VAC-system (Medela, Dietersheim, Germany) was inserted into the cavity of the former aneurysm. Due to a left pyothorax, re-intervention became necessary on day 20. After left lateral thoracotomy, a partial lung resection of the lower lobe and opening of the aneurysm from the left with insertion of VAC-system was performed. Exposed thoracic stent prosthesis was covered by a pediculated omentoplasty from the right, and thoracotomy was closed. Repeated lavage and re-insertion of the VAC-system from the left were performed for 2 weeks. Finally, the thoracic stent graft, which was exposed over a length of 8 cm, was covered with a pleura-parietalis flap plastic and the thoracotomy was closed.

After a stay of 7 weeks, the patient was transferred to a neurological rehabilitation clinic in significantly improved general condition and with no indication of renewed infection in the area of the thoracic stent prosthesis 5 months after primary diagnosis of AEF.

Elective esophageal reconstruction was performed in January 2018. The further course was prolonged because of anastomotic insufficiency of the gastroesophageal anastomosis. The patient wished to withdraw from therapy and passed away in April 2018.

## 3 Comment

The incidence of AEF following open surgery or TEVAR is reported to be 1.5–2,1% [2,3]. Main symptoms of AEF are hematemesis, fever or shock (septic and/or hemorrhagic) [2]. AEF without surgical treatment is usually fatal. Even with adequate surgical therapy, perioperative mortality is between 57 % and 64 % [2,4]. The gold standard for diagnosis is CT, possibly supplemented by EGD [2]. An established and generally accepted treatment approach does not exist. Graft explantation and radical surgical debridement is the therapy of choice for prosthetic infections [2,3]. Therapy alternatives are divided into four groups, i.e. conservative vs. esophageal stent vs. isolated esophagectomy vs. esophagectomy and aortic reconstruction. Conservative therapy resulted in death for all patients in previous studies [2,4].

Surgical treatment shows high morbidity and mortality rates. Kawamoto et al. were able to show a one- and five-year survival rate of 68.6 % and 42.9 %, respectively, in a retrospective study of 10 patients with primary and secondary AEF with multi-stage surgical therapy. Surgical therapy consisted of TEVAR or Re-TEVAR and subtotal esophagectomy as bridging therapy with subsequent aortic reconstruction with homograft or rifampicin-soaked Dacron prosthesis after four to 12 days and esophageal reconstruction in an infection-free interval after three to six months [5]. However, the surgical management and aortic reconstruction in primary AEF differ significantly from those in secondary AEF, making a study including both types difficult to interpret. In contrast, in a large multicenter study of pure secondary AEF after TEVAR, Czerny et al. showed one-year survival rates of 46 % with radical esophagectomy and immediate aortic reconstruction. Isolated esophagectomy, stent implantation or conservative therapy showed worse one-year survival rates of 43 %, 17 % and 0% [2].

For treatment of AEF with isolated esophagectomy, one-year survival rates of 43 %, 25 % and 0% were reported [[2], [3], [4]]. In eight patients examined by Luehr et al. with AEF after TEVAR, primary aortic reconstruction was omitted and esophagectomy alone was performed. One patient with a history of supra-aortal debranching before TEVAR and concomitant severe mediastinitis as well as multiple paraesophageal abscesses was treated with esophagectomy without aortic reconstruction. The patient died six days later from hypovolemic shock during rupture of the ascending aorta [3].

None of the cited studies describes a case as ours with a similar history of multiple vascular surgery interventions. The majority of patients with secondary AEF had received a simple TEVAR-procedure. AEF after aortic reconstruction of the entire aorta from the aortic arch to the aortic bifurcation has not yet been described. In comparison to previous literature, our case represents the complexity of the treatment of aorto-esophageal fistulas and its enormous demands on the interdisciplinary medical team. The standard treatment of thoracic vascular graft infections remains explantation and revascularization by reconstruction with homo-/xenograft or antibiotically pre-treated plastic prosthesis. Our report shows that in an emergency situation without other surgical options as in our case, it was possible to stabilize the patient through application of vacuum therapy in the infected area, with simultaneous esophagectomy, followed by secondary staged reconstruction with omentoplasty and pleura parietalis flap remaining the graft in situ. The long-term recovery process after this procedure remains uncertain, but the current case report showed preferable results for 5 months after primary diagnosis of the AEF.

## 4 Summary

Contrary to the generally accepted therapy recommendation for graft infection, we performed a multi-staged surgical therapy concept for aortoesophageal fistula including staged right and left thoracotomy with esophagectomy, vacuum therapy on the exposed stent prosthesis and subsequent graft coverage with omental and pleural flaps, followed by esophageal reconstruction. In an emergency situation where stent removal is not feasible, a multi-staged surgical procedure with leaving the thoracic stent graft in situ can lead to infection remediation.

## Declaration of Competing Interest

The authors declare no conflicts of interest.

## Ethical approval

Due to its character as a retrospective individual case report, this article does not meet DHHS definition of research and thus was exempt from ethical approval in our institution.Consent

Written informed consent was obtained from the patient for publication of this case report and accompanying images. A copy of the written consent is available for review by the Editor-in-Chief of this journal on request.

## Author contribution

Buerger M: Conceptualization, Methodology, Investigation, Writing – Original Draft,

Frese JP: Investigation, Writing - Review & Editing

Kapahnke: Writing - Review & Editing

Greiner A: Writing - Review & Editing, Supervision

## Registration of research studies

1 Name of the registry: N/A
2 Unique identifying number or registration ID: N/A
3 Hyperlink to your specific registration (must be publicly accessible and will be checked): N/A

## Guarantor

Andreas Greiner.

## Provenance and peer review

Not commissioned, externally peer-reviewed

## Funding

The authors declare no financial disclosures.
